# A biobank management model applicable to biomedical research

**DOI:** 10.1186/1472-6939-7-4

**Published:** 2006-04-06

**Authors:** Christiane Auray-Blais, Johane Patenaude

**Affiliations:** 1 Service of Genetics, Department of Pediatrics, Faculty of Medicine and Health Sciences, Université de Sherbrooke, 3001, 12^th ^Avenue North, Sherbrooke, Qc, J1H 5N4, Canada; 2 Department of Surgery, Faculty of Medicine and Health Sciences, Université de Sherbrooke, 3001, 12^th ^Avenue North, Sherbrooke, Qc, J1H 5N4, Canada

## Abstract

**Background:**

The work of Research Ethics Boards (REBs), especially when involving genetics research and biobanks, has become more challenging with the growth of biotechnology and biomedical research. Some REBs have even rejected research projects where the use of a biobank with coded samples was an integral part of the study, the greatest fear being the lack of participant protection and uncontrolled use of biological samples or related genetic data. The risks of discrimination and stigmatization are a recurrent issue. In light of the increasing interest in biomedical research and the resulting benefits to the health of participants, it is imperative that practical solutions be found to the problems associated with the management of biobanks: namely, protecting the integrity of the research participants, as well as guaranteeing the security and confidentiality of the participant's information.

**Methods:**

We aimed to devise a practical and efficient model for the management of biobanks in biomedical research where a medical archivist plays the pivotal role as a data-protection officer. The model had to reduce the burden placed on REBs responsible for the evaluation of genetics projects and, at the same time, maximize the protection of research participants.

**Results:**

The proposed model includes the following: 1) a means of protecting the information in biobanks, 2) offers ways to provide follow-up information requested about the participants, 3) protects the participant's confidentiality and 4) adequately deals with the ethical issues at stake in biobanking.

**Conclusion:**

Until a governmental governance body is established in Quebec to guarantee the protection of research participants and establish harmonized guidelines for the management of biobanks in medical research, it is definitely up to REBs to find solutions that the present lack of guidelines poses. The model presented in this article offers a practical solution on a day-to-day basis for REBs, as well as researchers by promoting an archivist to a pivotal role in the process. It assures protection of all participants who altruistically donate their samples to generate and improve knowledge for better diagnosis and medical treatment.

## Background

In Canada, Research Ethics Boards (REBs), also known as Research Ethics Committees (RECs), Institutional Review Boards (IRBs), and Ethical Review Boards (ERBs), have the mandate to approve, reject, propose modifications to, or terminate any proposed or ongoing research involving human subjects which is conducted within, or by members of, the institution, using the considerations set forth according to the Tri-Council Policy as the minimum standard. The authority of the REB being delegated through the institution's normal process of governance [1]. In Quebec, most REBs are under the administrative supervision of the Administrative Council of each medical centre. In fact, the everyday realities of life as a patient and health care provider or research subject and researcher are framed by the institutional settings of health care and research institutions [2].

REBs have, as primary concerns, the protection of the integrity of participants, the need for participants to receive simple but efficient information in order to grant an informed consent, the maintenance of confidentiality of participant's data, and decisions to inform the participants on the research being conducted and the transmission of research results (if applicable). It was accurately stated that "Institutional Review Boards have considerable experience in dealing with issues of recruitment, informed consent, confidentiality, and balancing risks and benefits. The context of commercial biobanking, however is relatively new" [3]. With the advancement of biotechnology and the growth in biomedical research, the role of REBs has become more complex, especially regarding research in genetics and related biobanks. The competence of REBs has been extended "to protocols potentially affecting not only the physical integrity, but also the identity (social and familial) of an individual, bringing psychological and spiritual aspects to the forefront" [4].

Considering that there are no specific regulations in Quebec, nor in the rest of Canada to control the creation and management of biobanks arising from institutional research projects, major ethical issues are on the rise and are often left to be managed by REBs and their affiliated institution. Nevertheless, the Research Ethics Committees appear to be appropriate bodies for evaluating research projects involving biobanks [4]. Recently, in Quebec, a newly-formed Institute for Populations, Ethics and Governance [5] has been created. It is a non-profit organization with the initial mission of coordinating large-scale research projects related to population genetics, genomics or proteomics taking place in member institutions. It had the initial mandate of managing the CARTaGENE project [6] and other biobanks in Quebec that wish to be so governed [7]. Therefore, every academic or research institution in Quebec with the above interests can become a member [5,7], but is not obligated to. Presently, there is no governmental governance body, in Quebec, nor in the rest of Canada, to manage biobanks stemming from academic or institutional research projects. In fact, there are no established harmonized guidelines that could give directions to REBs in managing biobanks in order to insure the protection of research participants.

Consequently, the following difficult questions about biobanks are emerging on a daily basis for REBs: 1) How can a REB deal with the growing demands for collecting, processing and storing biological samples and related information in genetic research projects? 2) When a participant in a research project gives his consent for the storage of samples in biobanks, how can the REB provide adequate privacy protection for him? 3) How is it possible to maintain the security and integrity of the research participants with the use of coded samples? (In fact, the demands for coded samples are becoming the rule compared to the use of anonymous samples just a few years ago). 4) How is it possible for REBs to protect the confidentiality and security of genetic information arising from these biobanks? 5) How can the storage of biological materials be managed for an indefinite period of time as often requested by sponsors, funding bodies and pharmaceutical companies? 6) What are the assurances given by REBs to research participants that their "informed consent" for the storage of their samples in biobanks is fully respected? 7) In the event of a discovery with clinical utility, how is it possible to recontact the research participants while respecting the confidentiality issue?

In light of the increasing interest in biomedical research [3,4,8,9] and the resulting benefits to the health of participants, it is imperative that practical solutions be found to the problems associated with the management of biobanks: namely, the protection of the integrity of the research participants, as well as guaranteeing the security and confidentiality of their information data. REBs also have a dual responsibility: firstly, to safeguard the dignity, rights, safety and well-being of research participants; secondly, to facilitate the good conduct of high quality research [10,11], without jeopardizing the integrity of research participants.

As members of institutional REBs for many years, we have noticed these growing concerns among our colleagues in trying to find answers to the questions raised above and the overall management of biobanks in genetics research. A recent Canadian survey among 43 REBs has clearly demonstrated the variability of answers and the difficulties arising from a fictional protocol involving the creation, use and management of biobanks in a genetic study. REBs were asked: Do you think that this protocol is ethically acceptable by your REB? 23% refused the protocol categorically, 26% wanted the protocol to be reviewed by the PI before deciding, 26% refused on condition of certain modifications, 14% stipulated that the protocol was not within their mandate, 9% accepted the protocol but under certain conditions and only 1% approved it without conditions [12]. In another study, 44 Canadian biomedical REBs agreed to review a mock research protocol in functional neuroimaging involving biobanks. The distribution of decisions made by the participating REBs is as follows: 68.2% rejected the protocol because of the creation of a biobank, 22.7% approved the protocol but under certain conditions, 6.8% approved unconditionally and 2.3% preferred not to answer. Interestingly, among the 68.2% of REBs who rejected the protocol, 37% expressed concerns about access to research subjects' medical records, 30% questioned the building of a database and 30% mentioned that the study involving biobanks did not fall under their mandate or area of expertise [13]. These two studies reaffirm the great variability of motives and criteria involved in the decision-making process for REBs. Their difficulties in managing research projects with biobanks often pose barriers to research.

REBs evaluating genetic research projects also have to deal with a dual problematic: first, the samples collected in the principal study are almost always coded by the principal investigator who remains the keyholder; second, there are growing demands from pharmaceutical companies and funding agencies to store residual samples. Also, there are requests for additional samples from the same participants and the storage of these additional samples in biobanks for an indefinite number of years. There is thus an increasing need to deploy adequate measures of confidentiality and protection of the integrity of the research participants without jeopardizing information that could benefit the entire research process and, in the end, be of major importance to the health of the participants.

In this article, we present a practical model for the management of biobanks in biomedical research, which could alleviate the burden placed on REBs who are trying to evaluate genetics projects and at the same time, maximize the protection of research participants. Consequently, we will address the protection of the information related to biobanks, the means to provide follow-up information about the participant without breaching confidentiality, the possibility of recontacting the participants in the event of a clinical utility stemming from the research with biobank samples and the ethical issues at stake for participants in biobanking. But first, let us define the terminology of "biobanks" and the "identifiability of samples" or biological materials stored in biobanks.

## Methods

### Biobanks

Biobanks refer to organized collections of biological samples and the data associated with them [14]. In the context of genetic research, biobanks are defined as a cluster of biological samples obtained from a group of identified individuals selected according to the clinical or biological characteristics of one or more members of the group, as well as any and all derivatives of these samples [4]. The American National Bioethics Advisory Commission defined a DNA bank as a "facility that stores extracted DNA, transformed cell lines, frozen blood or other tissue, or biological materials, for future DNA analysis". The same Commission defined a DNA databank as "a repository of genetic information obtained from the analysis of DNA, sometimes referred to as "DNA profiles". DNA sample collections are used for various purposes, such as for clinical, research and industrial uses [15]. Human biological samples in biobanks include organs (heart, liver, kidney, lung, pancreas, etc.), tissues, cells (somatic and gonadic), body fluids, hair, nails and body waste products [16]. Thus, biobanks are an important resource for identifying the causes and mechanisms of a large number of diseases, including, in particular, those that are widespread among the population [11]. Our ever greater understanding of the human genome is increasingly making it possible to determine the role, not only of environmental or lifestyle factors, but also of hereditary factors (genes) as the cause or disposition to disease (genetic epidemiology) [11].

Biobanks may be operated under the auspices of public sector institutions, such as university departments, individuals or private bodies – for example, pharmaceutical companies. Irrespective of the responsible institution, they may get funding from public or private sources [11]. In this article, we will address the problems facing biobanks created for institutional or academic research projects only, either publicly or privately funded.

### Identifiability of samples for research purposes

Ambiguity is often the case when referring to identifiability of samples for research purposes. A few years ago, the American Society of Human Genetics described four types of identification of samples for research purposes: a) anonymous: biological materials that were originally collected without identifiers and are impossible to link to their sources; b) anonymised: biological materials that were originally identified, but have been irreversibly stripped of all identifiers and are impossible to link to their sources; c) identifiable or coded or traceable: biological materials that are unidentified for research purposes, but can be linked to their sources through the use of a code. Decoding can only be done by the investigator or another member of the research team; d) identified: biological materials to which identifiers, such as name, patient number, or clear pedigree location, are attached and made available to the researchers [17]. Recently, the European Agency for the Evaluation of Medicinal Products gave some guidance in dealing with coded samples, adding the category "double-coded samples and results", where a first code is assigned to the sample and a second code is provided to link it to the results. The keycode linking the double coded pharmacogenomic samples and the information is kept by a third party. The research subject can only be linked with the sample or data obtained from it by bringing the two code keys together [18].

Even taking into account these precise definitions there is a tremendous variability in interpretations among REBs, regarding the four classes of identifiability [19]. So, in this article, we will be focusing mainly on issues concerning identifiable or coded samples in biobanking, also referred to as pseudonymized samples [11].

### Procedure for the collection of specimens in institutional research projects

Let us consider the procedure for the collection of biological samples in institutional research projects often requested in the scope of a multi-centric trial by funding bodies, such as pharmaceutical companies. After the approval of the research project by the REB and after a signed informed consent from the participants is obtained, the collection of biological samples is performed by the principal investigator's nurse coordinator at the clinical research centre affiliated to a medical centre where the patients are usually clinically treated and follow-up occurs. The samples are usually identified or coded with an alphanumeric code under the responsibility of the principal investigator (PI). Only the PI and a designated research coordinator have the keycode related to identifiers such as the name, date of birth, age, sex, disease, treatment, etc. The coded samples are then sent to the pharmaceutical or sponsoring company funding the project where haematological, biochemical, radiobiological, DNA analyses, etc. are done. Some research will look at the genotypic variation, others at the gene expression (at the RNA or protein level), and others still at the viral or tumoral genotype [20]. Often, in these multi-centric or international projects, samples from many collaborating countries are sent to a central lab where the analyses are performed by trained personnel using validated and uniform techniques.

In principle, it is not possible for the funding bodies to access the codes and thus find a link to the source. However, the American Society of Human Genetics (ASHG) recommended that investigators inform individuals that they cannot guarantee absolute confidentiality [21]. The ethics committee of the ASHG recommended that researchers "consider a way of coding samples by a third, independent party, who would keep the codes inaccessible unless there are specific circumstances in which the code needs to be broken" [15].

Also, what is often the case in pharmacogenetics research, a second specimen or residual specimen is requested for molecular genetics studies in order to better understand the disease, its response to drug and the possible adverse events. These samples are requested for storage in biobanks at the company's facilities, usually for an indefinite period of time. Currently, there is a growing need for coded second-specimen samples (in the past, such request samples were anonymous) and these requests involve serious ethical issues that must be dealt with by REBs.

### Ethical issues at stake for research participants in biobanking

There is a pluralism of values at stake as regards to patients, research participants, authorities, the medical profession, scientists and the general public concerning the storage and use of human tissue samples [22,23], the importance of which may vary according to the individual involved.

#### 1. Informed consent

It is generally accepted that the necessity of "Informed Consent" within the context of research is an absolute imperative [24]. In research involving biobanks, there are two main principles to be respected for the benefit of the participant who agrees to provide samples: obtaining his informed consent and determining the risk/benefit balance by the REB. Putting forward a legal framework for research involving biobanks, the German National Ethics Council, in a recent opinion, states as its first guiding principle, that "the central element of all regulatory proposals must be the donor's right of self-determination. This means that the collection of substances from his body and the gathering of personal data, for subsequent use in biobanks for the purposes of medical research, must be subject to the donor's consent. The consent is effective if the donor has the capacity to give consent, the consent is given voluntarily and the donor has been appropriately informed of the purposes, nature, significance and implications of the collection and use" [11]. After assessing the voluntary nature of the participation, it is the responsibility of the REBs to evaluate other important issues such as the possibility of stigmatization or discrimination in discrete subpopulations, traditional communities, such as indigenous peoples [11] (even if the research subjects samples are coded) and the possibility of benefit sharing of revenues that could be generated by the research.

It was recently mentioned that, "while it is possible to obtain informed consent to have one's blood, cells or tissue samples taken by researchers for a specific research project, the very intention of setting up such large data banks precludes giving informed consent for all the possible ways in which the information derived from that sample can be used for future research" [3]. But, given the speed of scientific development in the area of genetics and the vast spectrum of potential research hypotheses that may arise and can legitimately be addressed by such databanks, there is no way to predict possible future uses of donated samples [25]. It is up to the REB to "develop their own guidelines to evaluate research with stored biological materials" [20,26] to respect the consent of participants. An interesting recent survey revealed that the donor informed consent procedures in tissue-based research should be rethought: perhaps information about research objectives is seen more as a service than a safeguard (i.e., is nice to have but not important), which would imply that bioethicists should work to ensure safeguards other than informed consent. In fact, it has been suggested to focus attention on the establishment of tissue-trustee infrastructures, rather than consent issues, to ensure the confidence of the donating public [27].

Nevertheless, it is essential for REBs to put in place means for the logistics involved in the uses of donor's specimens stored in biobanks for the purposes of medical research.

#### 2. Confidentiality issue

Areas of concern related to confidentiality in the context of commercial biobanking include handling of identifiers, physical and other kinds of security, and transfer of samples and information [28,29]. The breach of confidentiality resulting from the mishandling of personal information is an important issue in pharmagenomics (and pharmacogenetics). This informational risk is due to the personal, familial, and social nature of genetic information as well as its potential to discriminate and stigmatize [20]. Points to consider by researchers and Institutional Review Boards (IRBs) have recently been suggested in determining various levels of confidentiality, within the framework of pharmacogenomics research where researchers decide the level of protection best suited for their research protocols [20]. Clearly, both researchers and IRBs will need to work in unison, on a case by case basis, with participant confidentiality as a primary concern. In the end, IRBs will have to evaluate the final acceptable level of confidentiality.

One of the guiding principles of the German National Ethical Council is that donors must be protected by an obligation of confidentiality on the part of all involved in the establishment and use of biobanks. When not provided by law, this obligation of confidentiality must be imposed by the institution itself, either in its statutes or by contract [11]. A study to determine if REBs provided ethical guidance in research involving stored biological specimens revealed that, although most REBs mention the confidentiality issue, "fewer than 30% suggested steps for protecting confidentiality" [26]. Hence, it is up to each REB to be vigilant in respecting the statutes of its own institution and also in establishing protocols and guidelines (if not already established) to ensure protection of the genetic data information of the research participants.

#### 3. Privacy issue and protection of information

A recent survey in Sweden revealed that in spite of widespread bioethical debate about the presumed sensitivity of genotype information, management of medical records and clinical data appear to remain of great concern to the donating public [30]. Guidelines developed by the Ethics Committee of the Swedish Medical Research Council stress the importance of protecting individual data and advise that codes linking data held in a biobank to an individual should be kept within a public institution such as a university or medical authority [31]. If inadequate attention is paid to security, unauthorized individuals can access electronically stored information and use it for illicit purposes. As a result people are increasingly worried about their privacy and want stricter control over who has access to information about them and under what conditions [19,32]. Issues of privacy have become entangled with bioinformatics as, increasingly, we rely on technology rather than on human beings to resolve privacy issues [33]. Fears of discrimination by employers and insurers are definitely of increasing importance for participants in genetic research. Many may fear that their genetic information could be shared with third parties (insurers, employers), who sometimes require that the individual provide a general release of his medical records or information relating to his participation in research projects.

It is essential that operational rules be established by REBs so that the conditions of access to biobanks are clearly determined and are acceptable to the research participants.

## Results

### A proposed biobank management model for institutional research

**Figure 1:**
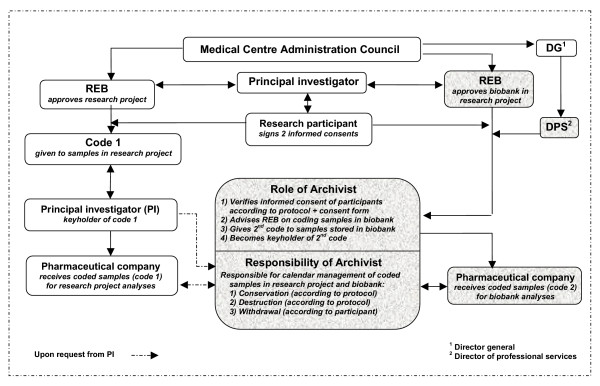
Schematic diagram of management model for biobanks. (Shaded areas are for biobank management)

We propose a model for the management of biobanks in biomedical research. Figure 1 represents a schematic description of this model, taking into account the administration levels encountered in a medical centre. This model is based on the use of two codes, each being specifically attributed to a definite part of the study: **Part 1**: collection of samples with a specific code (code 1) for the research project and **Part 2**: collection of samples with a specific code (code 2) to be stored in a biobank for genetic analyses.

#### Part 1 (Code 1)

After approval of the research project by the REB and informed consent obtained from willing participants, samples are collected and coded. For this main study, an alphanumeric code (code 1) is given to each sample. The principal investigator of the project, at the institution, is responsible for keycode no. 1. The coded samples are then usually sent to the pharmaceutical company for analyses, as previously described.

Presently, the PI is the one responsible for managing the schedule of conservation, as well as the destruction and withdrawal of the coded samples. In the model presented herein, the PI has the option of bestowing this responsibility on a medical archivist, who has to respect the law and regulations applicable to managing archives in medical centres, the archivist being a key person in maintaining the confidentiality [34,35]. Since clinical researchers, in an academic institution, are also practicing physicians, teaching, as well as doing research in many projects (multi-centric or not), they can go on sabbatical, be on vacation, take sick-leave and eventually retire, it could probably be a very interesting option for them to have a professional medical archivist at their medical centre taking care of this very important task of managing coded samples from research projects. Since coded research samples may be kept for a period of time that may vary from a few weeks to many years, depending on the protocol of the funding bodies, a schedule of conservation and management of samples could be established in collaboration with the researcher, upon acceptance of this procedure. Therefore, the archivist would receive the keycode for that particular research project, and effectively manage the samples on a daily basis, always in close cooperation with the PI. We thus propose a collaboration between PI and archivist to maintain an efficient way of managing coded samples necessary for the main study.

#### Part 2 (Code 2)

The second part of the study represents the collection of samples for the biobank. The responsibilities and obligations of the PI can be divided into 3 important sections in this biobank part of the project: 1) to evaluate whether the ethics principles involved are respected in the creation of the biobank, as well as the need, reasons, objectives and scientific importance for biobanking in the first place; 2) to explain the preceding section to the REB and to provide answers to their questions; 3) upon acceptance of the creation of the biobank by the REB, the PI must explain to the research participants, in the most comprehensible way, the scientific importance of giving their biological samples to the biobank and all circumstances likely to be relevant to their decision to grant or refuse the biobanking, such as the need, the purposes, the nature, the scope and duration of the proposed use, the extent and conditions of a possible transfer of samples and data, the form of data storage and linkage, the possibility of withdrawal of consent at any time, the commercial prospects, the benefit sharing [11], the possibility of being recontacted (or not) in the event of a discovery of medical utility and inform them about the role of the archivist, as the person in charge of the management of the biobank. These are crucial responsibilities and obligations that the PI can not delegate to the archivist.

As mentioned previously, REBs must approve biobanking and set detailed conditions for access to the biobanks and their use. The research participants, after receiving complete information about the biobank and responses to their possible questions, sign a separate consent form in which they agree to donate their biological samples to the biobank. In the proposed model, an alphanumeric code is then attributed, by a medical archivist, to each sample (code 2). The samples are then sent to the central laboratory of the company, where the analyses are performed. The only person having the keycode no. 2 is the archivist. The archivist, as a third party, has the role of a data-protection officer [11,36,37] or trustee [28] who is independent from the research project. However, this role is relatively different from an honest broker system [38]: first, the archivist is appointed by the institution, under the responsibility of the Director of Professional Services of the medical centre and in accordance with the Clinical Research Centre, where the research is performed, not the REB (as often seen with the honest broker system). Secondly, the educational training, role, duty, obligations and responsibilities of the medical archivists are different, in comparison to an honest broker, who can be a nurse, a clinical clerk and/or a computer software system, etc. Also, the archivist has been trained to adequately manage the schedule of retention of charts in a medical centre, so it would be easy to apply the same principles to coded samples in a research project (conservation, destruction, withdrawal). The archivist will ensure the protection of data and supervise the biobank. Hence, it is the responsibility of the archivist to ensure good compliance with the legal requirements applicable when handling personal information and data [34,35]. Regarding ethics principles involved, the archivist has the obligation to respect the Code of Ethics and politics of confidentiality instigated by the medical centre, and legally, the different laws applicable in Quebec, such as the Archives Act and Regulations [34,35], the Health services and social services Act [39], the *Civil Code of Quebec *[40], the Access to documents held by public bodies and the protection of personal information Act [41]. The role of the archivist is thus under the governance of ethical and legal frameworks, that are oriented towards the protection of the integrity of patients, who can also be research participants in biomedical research.

Furthermore, it was recently recommended that the researcher can not have access to keycode no. 2 "so that he cannot by himself link them to the person concerned. In this case, as in that of anonymization, the researchers lack the possibility of relating samples and data to their donors" [11,36]. The reality of biobanks is such that the role of the PI is very limited. He is not responsible for the genetics research being done at the pharmaceutical company, nor is he implicated in the direction that the research will take. He may however be asked to give information regarding the medical condition of the research participant, the side effects of a specific drug, etc. The role of the archivist as a data-protector is essential in coordinating and answering these particular questions.

Furthermore, in the event of a clinical utility stemming from the research with biobank samples, it could be important to recontact the research participants. The pharmaceutical companies and funding agencies would then have to contact the PI to reveal the importance of the discovery and the need to recontact the participants. After confirming the importance of the discovery and the need of recontacting the participants, the PI would then resubmit this new demand to the REB of his institution. The REB would have the responsibility to evaluate the merits of recontact, the proportionality of risks and benefits involved and even in certain cases, the probable social consequences that could arise. If there is an approval by the REB to recontact the participants, the REB would then inform the archivist who would verify if the participants have previously agreed in their consent form to be recontacted. If so, the archivist could proceed to recontact the participants and inform them of the particular beneficial situation. A meeting with the PI would then be scheduled for further explanation.

The archivist plays an essential role in this model, since he/she holds the research protocol and the signed consent form from the participant, in order to verify that his wishes are in accord with the agreement established at the start of the project. Apart from being the keyholder of coding no. 2, the archivist will also be responsible for the schedule of conservation, the destruction or for the withdrawal of specimens. Also, a participant who decides to terminate his participation in the project will have the opportunity to withdraw at any time from the study, the samples being coded. In addition, if the pharmaceutical company decides to ask questions relating to the clinical aspects of the project, such as the response of the participant to a specific drug, his current medical situation, etc., the archivist will be able to answer the questions, while ensuring the confidentiality of data.

## Discussion

The model for managing biobanks presented herein is simple, efficient, and offers interesting practical possibilities for Research Ethics Boards, the researcher and the funding bodies, and mostly, is of major importance for the protection of the integrity of the research participant. The proposed model is novel regarding the fact that it does not require new personnel, committees or training of any kind. In fact, it favours the use of trained medical archivists, who are part of an infrastructure already in place in a medical centre, and who have proven to be efficient throughout the years in managing charts of patients, under specific laws and a strict code of ethics, to assure the management of the research biobanks. It also answers questions mentioned in the introduction, regarding the day-to-day collection, storage and processing of samples in biobanks. It insures a high degree of confidentiality, security of data information and protection of the integrity of the participants while using coded samples in the biobank that are distinct from that in the main research project. It offers the possibility of managing stored biological samples for a prolonged period of time, allowing research participants to withdraw their samples at any time, by a simple request to the archivist. It also favours the possibility for the funding body of asking information about the medical condition of the participant taking part in the research project, without breaching confidentiality. If a discovery of a medical utility arises, it is also feasible under this model to recontact the research participants.

These aspects are of major importance to the REB enabling it to ensure better protection of the integrity of the research participant. Evidently, the use of two non-related keycodes (keycodes 1 and 2), where the researcher has no access to keycode 2 for the samples and related information for the biobanks (he cannot by himself tie them to the participant), favours the highest level of confidentiality and security of the biobank data for the participant.

The model also respects the informed consent of the research participant, while having a health-care professional, such as an archivist or data-protection officer, in charge of the biobank and to whom they have entrusted their biological samples, as well as the related information and data. This situation will promote an evolutive, dynamic communication process between the funding body and the data-protection officer, facilitating exchanges of information. In the overall procedure, this model will also permit the sharing of valuable medical information on the participant, since the keycode offers a link to the biobank analyses, this may enhance the repercussions of the research project. The participant can also decide to withdraw his consent for the use of his samples and related data at any time, simply by asking the archivist. It also offers the possibility for the funding body to provide results of the research done to the participants, if they agreed to this in the first place in their informed consent.

## Conclusion

As long time members of REBs, we have been able to assess the burden on REBs in trying to evaluate research projects involving the use of storage samples in biobanks. The recurrent problems concerned the assurance of protection of the integrity of the participant, as well as the confidentiality and security of related data. We have presented two surveys clearly demonstrating the difficulties for REBs to manage research projects with biobanks and the high percentage of rejection of protocols involving the use of biobanks where coded samples were an integral part of the study. The greatest fear encountered being the lack of participant protection and uncontrolled use of biological samples or related genetic data. The risks of discrimination and stigmatization being a recurrent issue.

Until a governmental governance body is established in Quebec to guarantee the protection of research participants and establish harmonized guidelines for the management of biobanks in medical research, it is definitely up to REBs to find solutions. We therefore think that this model of biobanks management in medical research will offer an interesting solution on a day-to-day basis for REBs (hopefully in many countries), as well as for researchers, and in the end, assure protection of all participants who altruistically donate their samples to generate and improve knowledge for better diagnosis and medical treatment.

## Competing interests

The author(s) declare that they have no competing interests.

## Authors' contributions

CAB contributed substantially to the research, conception, design and development of the biobank management model. JP made substantial contribution to the development of the biobank management model. The two co-authors contributed to the drafting of the manuscript and have approved this final version in view of its publication.

## Pre-publication history

The pre-publication history for this paper can be accessed here:


